# Chylous Ascites Pointing Toward an Internal Hernia in the Setting of Roux-en-Y Gastric Bypass: A Case Report

**DOI:** 10.7759/cureus.33857

**Published:** 2023-01-17

**Authors:** Haider Sami, Hassan Raza, Beenish Fatima Zia, Ahmer Ashraf, Ammar Khawar

**Affiliations:** 1 Internal Medicine, Lady Reading Hospital, Peshawar, PAK; 2 Medicine, Lahore Medical and Dental College, Lahore, PAK; 3 Medicine, Fatima Memorial Hospital College of Medicine and Dentistry, Lahore, PAK; 4 Internal Medicine, Mayo Hospital, Lahore, PAK

**Keywords:** mesenteric internal hernia, internal hernia, chylous ascites, roux-en y, laparoscopic roux-en-y gastric bypass

## Abstract

Chylous ascites is a rare but significant complication of a variety of surgical procedures. It is an uncommon complication of laparoscopic Roux-en-Y gastric bypass (LRGYB). The underlying etiology is assumed to be an internal hernia, in which the hernia causes lymphatic channel engorgement and lymphatic extravasation. We present the case of a 34-year-old male who had a history of LRGYB a year back and had been experiencing gradually worsening, colicky abdominal pain radiating to the right flank for the last 24 hours. Laparoscopic exploration revealed chylous ascites due to internal herniation owing to the complication of LRYGB. Classic signs of internal hernias such as mesenteric swirl were absent on the computed tomography scan of the abdomen.

## Introduction

Chylous ascites is a creamy-colored intra-abdominal accumulation of fluid with a triglyceride concentration of more than 110 mg/dL [1]. It is a rare complication of laparoscopic Roux-en-Y gastric bypass (LRGYB). Roux-en-Y gastric bypass (RYGB) is one of the most routinely used surgical procedures even in adolescents. The procedure entails the development of a tiny, vertically oriented gastric pouch with a volume of less than 30 mL. The upper pouch is entirely split by the gastric remnant, which is anastomosed to the jejunum 30-75 cm distally to the Treitz ligament, effectively excluding a stomach component from further endoscopic investigation [2].

The underlying etiology of chylous ascites in LRGYB is assumed to be an internal hernia, in which the hernia causes lymphatic channel engorgement and lymphatic extravasation [3]. Classic mesenteric swirl or whirlpool sign is frequently observed in a computed tomography (CT) scan of the abdomen in such cases indicating an internal hernia [4]. We present a unique case of chyloperitoneum owing to the complication of LRYGB in which the classic mesenteric swirl, suggesting an internal hernia, was absent on the abdominal CT scan.

## Case presentation

A 34-year-old male with a history of LRYGB a year back presented with a complaint of gradual onset, colicky, worsening, mild to severe generalized abdominal pain with radiation to the right flank for the last 24 hours. The pain was associated with nausea and anorexia without vomiting. There were no modifying factors, prior episodes, fever or sick contacts. Abdominal tenderness was present, and vital signs were normal. There were no significant findings on general systemic examination. Differential diagnosis included kidney stones, diverticulitis, urinary tract infections, appendicitis, diverticulitis, small bowel obstruction, and midgut volvulus.

**Figure 1:**
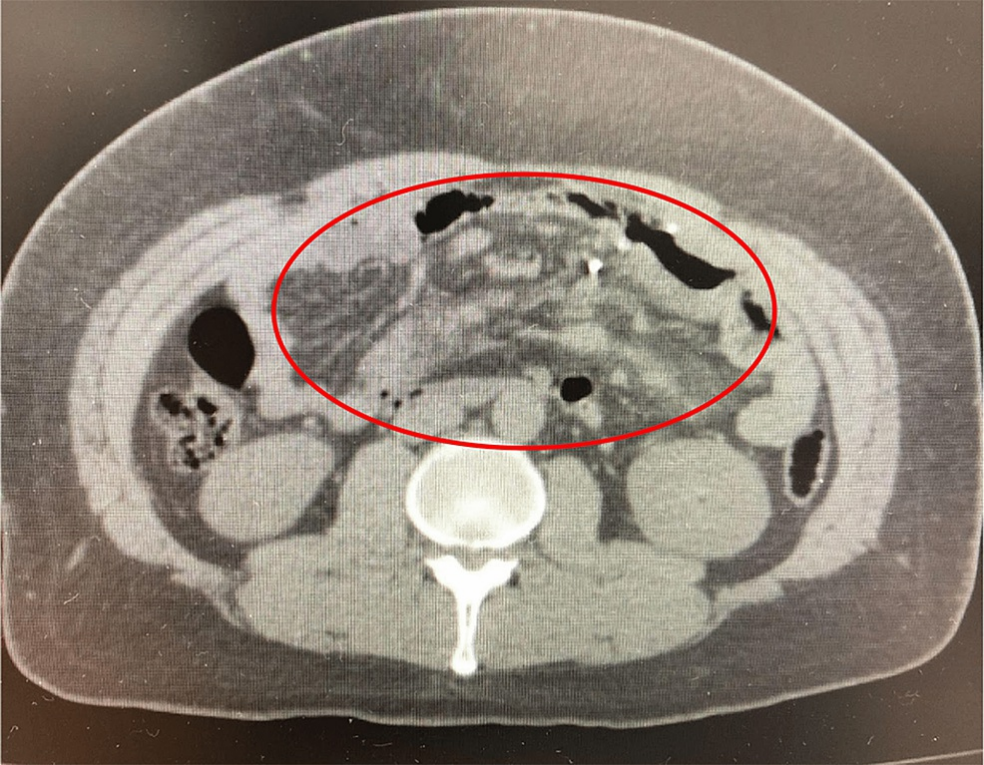
Transverse CT scan of abdomen showing misty mesentery appearance. CT: Computed tomography

CT scan of the abdomen suggested prior gastric bypass surgery. Rotation of the mesentery root with a misty mesentery appearance was observed. There was no evidence of bowel obstruction to suggest midgut volvulus (Figure 1).

**Figure 2:**
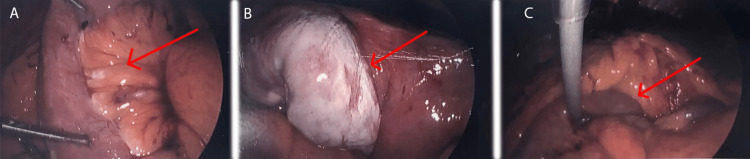
Laparoscopic examination of abdomen (A) inflamed Roux limb, (B) jejunojejunostomy, and (C) proximal Roux limb going retrocolic.

There was no evidence of obstructive nephropathy on ultrasonography. Laparoscopic exploration suspecting bowel obstruction revealed mild purulence in the right paracolic gutter and perihepatic space (Figure 2). The space posterior to the left lobe of the liver around the gastrojejunal anastomosis was covered with dense adhesions, but no inflammation or purulence was observed in the left upper quadrant. The small bowel was examined along the common channel, and also along the Roux limb. There was no evidence of internal hernia, and the bowel did not appear to have any unusual twisting more than what is customary with the Roux-en-Y configuration. There was a 20cm segment of the distal Roux limb which appeared to be surrounded on its serosa and its mesentery by whitish inflammatory exudate indicating chyloperitoneum. This segment of the bowel extended to the jejunojejunostomy, and there was not much length between this segment and the retrocolic opening for the Roux limb.

The bowel did not appear to be necrotic at any portion. Given its viability, the decision was made not to resect this part of the Roux limb and jeopardize the continuity of the alimentary tract. A peritoneal drain was inserted to remove the fluid collected in the peritoneum. The patient was given pain control after exploratory laparoscopy and then transferred for higher care.

## Discussion

Chyloperitoneum is the medical term for the accumulation of lymphatic fluid in the peritoneum as a result of a leak or rupture in the lymphatic system. Three things are thought to be responsible for this buildup: (1) When a tumor obstructs a vessel, the vessel may burst or exude fluid through dilated lymphatic channel walls. These persons frequently have enteropathies that lose protein, (2) a fistula develops between the lymphatic vessels and the peritoneum or mesentery as a result of adhesions, injury, or surgery, and (3) retroperitoneal massive lymphatic leakage, commonly brought on by congenital diseases, with or without fistula symptoms [3].

Prior to the introduction of Roux-en-Y gastric bypass surgery, the paraduodenal hernia was the most prevalent internal hernia, accounting for more than half of all occurrences [5]. However, due to the rising popularity of bariatric surgery in the mid-2000s, postsurgical internal hernias have become considerably more prevalent [6,7]. Internal hernias occur in between 0.2 and 11% of all patients after LRYGB, with a mortality risk of roughly 1.6% [8]. Hernias that develop after LRYGB surgery provide a significant risk of torsion and intestinal ischemia, necessitating early detection and surgical treatment [9]. Unfortunately, despite developments in multi-detector CT equipment and the widespread use of multi-planar reconstruction techniques, radiographic detection of any internal hernia remains tough, made much more difficult by the intricacy of post-bariatric-surgery anatomy [8].

The typical RYGB surgical method starts with the gastric fundus being used to build a tiny gastric pouch while the excluded stomach is kept intact [10,11]. To create the Roux limb (also known as the efferent or alimentary limb), the jejunum is divided 25-50 cm distal to the Treitz ligament, and the distal piece is elevated up and anastomosed to the gastric remnant. The Roux limb can be carried up over the transverse colon mesentery (antecolic) or through it (retrocolic). Finally, the biliopancreatic or afferent limb is anastomosed in a side-to-side way 75-150 cm distal to the G-J anastomosis (the enteroenterostomy or J-J anastomosis) [12]. In our case, a retrocolic Roux limb was observed.

Internal hernia symptoms are ambiguous and non-specific, making diagnosis challenging. When they initially come, the majority of patients report hazy gastrointestinal symptoms such as nausea and vomiting [13]. There is no rise in laboratory tests (such as WBC, renal function, liver function, or lactic acidosis) until there is an infarction or perforation [14].

After LRYGB, a CT scan has proven to be an effective tool for diagnosing internal hernias. There are certain CT scan imaging findings that, if present, raise suspicion for an internal hernia even if distorted anatomy makes it challenging to establish the diagnosis. Seven symptoms that can indicate an internal hernia in a CT scan were studied by Lockhart et al. in 2007 for their specificity and sensitivity [15]. These signs included the swirl sign, superior mesenteric vein strangulation, engorged mesenteric vein and oedema, enlarged lymph node, ascites, mushroom sign, and hurricane eye. The CT scans of 18 patients who had LRYGB and developed an internal hernia were contrasted with the CT scans of 18 patients who underwent LRYGB and developed an internal hernia but underwent no surgical intervention. They discovered that the swirl sign had a sensitivity of 61-94% and a specificity of 67-89% when compared to the control group [15].

This instance shows that chylous ascites is a sign of an internal herniation brought on by a mesenteric defect after an LRYGB. These instances might present hazy or misleading symptoms. In post-LRYGB patients, doctors should have a high index of suspicion for internal hernias, even if patients only have mild to moderate symptoms of colicky abdominal pain when they first present. If an internal hernia is detected too late, the chance of developing an acute small intestinal obstruction increases. In such cases, bariatric surgery or laparoscopic exploration should be considered since it is the most accurate and sensitive diagnostic procedure available, as compared to imaging like a CT scan, which is inadequate for diagnosis [16]. In the presented case, the absence of the classic signs of internal herniation on abdominal CT scan clearly supports this fact.

## Conclusions

In conclusion, our case presented a patient who, a year after undergoing an LRYGB, acquired chylous ascites but no internal herniation was present. Chylous ascites in adults is most frequently caused by cancer, although it can also manifest in an unusual way due to an internal herniation brought on by a post-LRYGB mesenteric abnormality. In many cases, chylous ascites presents with relatively mild and ambiguous symptoms, which may lead to a different differential diagnosis than a probable internal hernia. As a result, all LRYGB patients who present with minor abdominal symptoms with chylous ascites should not be suspected of an internal hernia and should be treated accordingly.
